# Finger Pain as an Uncommon Primary Manifestation of Lung Carcinoma

**DOI:** 10.3390/diagnostics13050901

**Published:** 2023-02-27

**Authors:** Adrien Holzgreve, Hans Roland Dürr, Axel Stäbler, Mathias Kaemmerer, Lena M. Unterrainer, Amanda Tufman, Farkhad Manapov, Wolfgang G. Kunz, Marcus Unterrainer

**Affiliations:** 1 Department of Nuclear Medicine, University Hospital, LMU Munich, 81377 Munich, Germany; 2 Musculoskeletal Oncology, Department of Orthopaedics and Trauma Surgery, University Hospital, LMU Munich, 81377 Munich, Germany; 3 Radiologische Praxis München Großhadern, 81377 Munich, Germany; 4 Department of Internal Medicine V, University Hospital, LMU Munich, 81377 Munich, Germany; 5 Department of Radiation Oncology, University Hospital, LMU Munich, 81377 Munich, Germany; 6 Department of Radiology, University Hospital, LMU Munich, 81377 Munich, Germany; 7 DIE RADIOLOGIE, 80331 Munich, Germany

**Keywords:** magnetic resonance imaging (MRI), non-small cell lung carcinoma (NSCLC), rare symptom, unusual presentation

## Abstract

A 54-year-old patient presented with progressive pain for one month in the second finger of the right hand with an emphasis on the proximal interphalangeal (PIP) joint. Subsequent magnetic resonance imaging (MRI) showed a diffuse intraosseous lesion at the base of the middle phalanx with destruction of the cortical bone and extraosseous soft tissue. An expansively growing chondromatous bone tumor, e.g., a chondrosarcoma, was suspected. After incisional biopsy, the pathologic findings finally revealed, surprisingly, a metastasis of a poorly differentiated non-small cell adenocarcinoma of the lung. This case illustrates a rare but important differential diagnosis for painful finger lesions.

## Detailed Figure Legend

**Figure 1:**
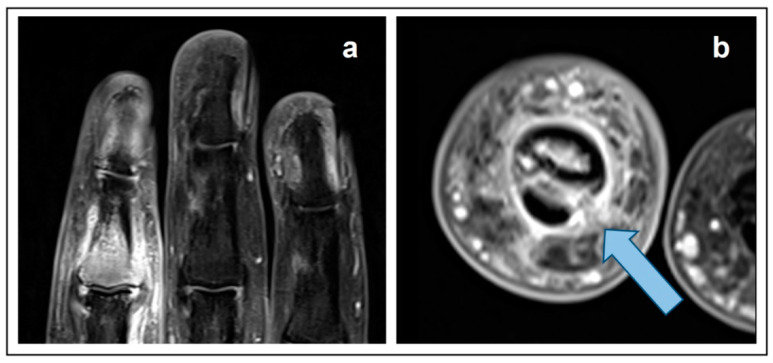
T1-weighted fat-saturated, contrast-enhanced MRI. (**a**) coronal view. (**b**) axial view.

A 54-year-old patient presented with progressive pain for one month and mild swelling in the second finger of the right hand with an emphasis on the proximal interphalangeal (PIP) joint. Subsequent 3T magnetic resonance imaging (MRI) of the right hand in T1-weighted fat-saturated sequence after administration of 20 mL Gd-DOTA showed a contrast-enhancing diffuse intraosseous lesion at the base of the middle phalanx with destruction of the cortical bone and surrounding extraosseous soft tissue (Figure 1a). The axial view (Figure 1b) revealed ulnar infiltration of the ligaments of the articular capsule (arrow). Based on the imaging findings, an expansively growing chondromatous bone tumor, e.g., a chondrosarcoma, was suspected. After incisional biopsy, the pathologic findings finally revealed, surprisingly, a metastasis of a poorly differentiated non-small cell adenocarcinoma of the lung.

**Figure 2:**
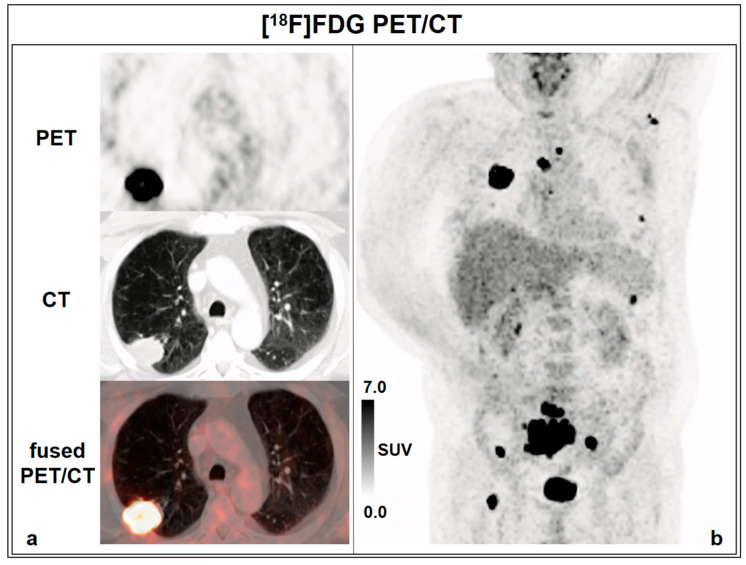
[^18^F]FDG PET/CT. (**a**) axial view. (**b**) maximum intensity projection (MIP).

The patient stopped smoking 12 years ago (after 20–30 pack years); there was no positive family history, no B symptoms (i.e., fever, night sweats, unintentional weight loss), occasional cough, no sputum, and no hemoptysis. Staging by positron emission tomography/computed tomography (PET/CT) with 316 MBq [^18^F]FDG revealed a primary tumor in the upper lobe of the right lung (Figure 2a) and multiple lymph node, soft tissue, and bone metastases (Figure 2b); cranial MRI revealed a brain metastasis.

In contrast to this case, typical initial symptoms of lung carcinoma are respiratory symptoms including cough, dyspnoea or haemoptysis, but not bone pain [1]. Overall, the occurrence of metastases in the acral parts of the body is a rare condition [2,3]. Amongst acro-metastases, lung cancer was reported to be the most common cause, with one third of cases [4]. The imaging findings of peripheral bone metastases, however, are variable and a robust differentiation from chondrosarcoma or other malignant bone and soft tissue tumors is not regularly possible using MRI alone [5]. Typically, medullary chondrosarcomas present with a lobular growth pattern at the margins and display a heterogeneous signal behavior across the sequences in MRI, partly due to diverse matrix mineralization and areas of entrapped yellow marrow [6].

These images illustrate the findings in a rare but important differential diagnosis for painful finger lesions and underline the value of timely histological confirmation, especially in the case of inconclusive imaging findings. Further, they recall that patients with advanced stage cancer may be either asymptomatic or symptomatic with an unusual pattern.
